# Cognitive training improves executive function and self-efficacy in young women with chronic stroke: a pilot study

**DOI:** 10.3389/fnhum.2025.1677642

**Published:** 2025-10-13

**Authors:** Michelle S. Scheffler, Asha K. Vas, Catherine Cooper Hay, Lisa Griggs-Stapleton, Lori G. Cook

**Affiliations:** ^1^Texas Woman's University, Denton, TX, United States; ^2^The University of Texas at Dallas, Richardson, TX, United States

**Keywords:** executive function, daily living skills, young women, chronic stroke, cognitive training

## Abstract

**Introduction:**

Young women are increasingly affected by stroke and often experience persistent executive function deficits that impact global functioning. The purpose of this pilot study was to evaluate the feasibility and effectiveness of a strategy-based cognitive training program (Strategic Memory Advanced Reasoning Training) to improve executive function and related outcomes in young women with stroke.

**Methods:**

Eight women with chronic-stage stroke (age: *M* = 38.75 years; *SD* = ± 8.78) and eight age- and education-matched controls (age: *M* = 35.75 years; *SD* ± 7.71) completed 10 sessions of SMART over 5 weeks, with pre- and post- training assessments. Outcomes included measures of executive function (subtests of the BrainHealth Index), daily living skills (Cognitive Self-Efficacy Questionnaire, Daily Living Questionnaire, and Community Integration Questionnaire), and psychosocial functioning (Depression, Anxiety, and Stress Scale-21), as well as feasibility/goodness of fit of the program (Acceptability of Intervention Measure, Intervention Appropriateness Measure, and Feasibility of Intervention Measure).

**Results:**

Following SMART, participants with stroke demonstrated improvements in aspects of executive function (including abstraction, strategic memory, and fluency of ideas), cognitive self-efficacy, and stress. Control participants also demonstrated gains, particularly in cognitive self-efficacy. Feasibility was rated highly by participants with stroke.

**Discussion:**

Findings support SMART as a promising intervention for enhancing cognitive and functional outcomes in young women with stroke, warranting further large-scale investigation.

## 1 Introduction

Nearly 800,000 individuals are estimated to be affected by stroke in the United States annually (NICHD, 2022). Although the prevalence of stroke is highest in older adults (aged 65 and older), occurrence in younger individuals is increasing, with the potential for long-lasting effects impacting their well-being for the rest of their lives. Women are increasingly experiencing strokes at a younger age (Bushnell et al., 2014) and there is a higher incidence of stroke in women than in men for ages 25–44 years (Leppert et al., 2020). While decreased physical activity and hypertension are recognized as key risk factors in strokes occurring in all age groups (Aigner et al., 2017), other risk factors recently cited as particularly prevalent in young women included pregnancy, post-partum, use of oral contraception, and migraine history (Thomas et al., 2021). Additionally, young women may present with non-modifiable risk factors such as clotting disorders, systemic diseases, and migraines (van Alebeek et al., 2018).

Young women often find themselves entrenched in the instrumental activities of daily living and social participation of early adult life, which include working outside the home, pursuing an education or career, providing child or elder care, maintaining relationships, and managing various household responsibilities (Perry-Jenkins and Gerstel, 2020). The cognitive demand, particularly as it pertains to executive function, may impact young women's ability to successfully and satisfactorily complete these higher-level daily living tasks. In turn, this leads to decreased satisfaction with roles, routines, and activities, and contributes to secondary challenges such as anxiety, depression (Ignacio et al., 2022) and decreased social participation.

Executive function is a vital group of cognitive processes necessary to control human goal directed behavior and appropriately respond to novel situations, which includes memory, planning, initiation, organization, inhibition, problem solving, and self-awareness (Smith and Jonides, 1999; Lux, 2007; Chung et al., 2013). Executive function deficits can lead to a decrease in participation in meaningful and necessary activities (Mole and Demeyere, 2020). Additionally, female sex and impairments in executive function have been identified as predictors of decreased participation outcomes post stroke (Adamit et al., 2015; de Graaf et al., 2018).

For women, evidence supports decreased life satisfaction post stroke, particularly in vocational and financial aspects of life (Röding et al., 2010). Similarly, stroke has a profound impact on women's relationships, sense of self, work, family responsibilities, and social activities (Leahy et al., 2016; Alenljung et al., 2019). These occurrences contribute to social isolation, decreased community integration, and psychosocial complications (Mukherjee et al., 2006; Viscogliosi et al., 2011). Therefore, positive change in executive function following stroke is critical to improve daily living skills, self-confidence, self-efficacy, social integration, and psychosocial factors in young women with stroke.

Against this backdrop, selection of an effective and salient cognitive training program remains critical. The strategy-based programs Cognitive Orientation to daily Occupational Performance (CO-OP) and Functional and Cognitive Occupational Therapy Intervention (FaCoT) and skills-based Computer-based Executive Function training (COMPUTER training) have demonstrated improved clinical performance for adults post stroke but did not exclusively reflect changes in young individuals with acquired brain injury (Poulin et al., 2017; Adamit et al., 2021). The Strategic Memory Advanced Reasoning Training (SMART) program is an evidence-based approach that emphasizes a systematic, strategy based approach to target executive function (Gamino et al., 2014; Samuelson et al., 2020; Vas et al., 2020; Young et al., 2021). SMART focuses on three main strategies: (a) strategic attention to key information, (b) integrated, gist-based reasoning for problem solving and daily scenarios, and (c) mental flexibility (Chapman et al., 2021). Regular application of SMART strategies can positively affect abstraction, fluency of ideas, strategic memory (ability to recall key details and information), and gist-based reasoning—key cognitive skills targeted by the training. SMART has also been effectively used in a telehealth format both for training and assessment components (Vas et al., 2016; Cook et al., 2020; Chapman et al., 2021; Laane et al., 2023). SMART has demonstrated positive outcomes in healthy adults, young adults with mild traumatic brain injury, and in older adults with stroke or brain injury (Vas et al., 2011; Cook et al., 2014; Chapman et al., 2017; Vas et al., 2018; Cook et al., 2020; Vas et al., 2020). Neuroimaging metrics have also demonstrated that SMART promotes neuroplasticity in adults (Vas et al., 2016; Chapman et al., 2017; Han et al., 2018).

The purpose of this pilot study was to determine the feasibility and effects of SMART on executive function and daily life function in young women with stroke. The aims of the study were as follows:

- To compare changes in executive function, daily living skills, and psychosocial function (1) in young women with stroke pre vs. post SMART and (2) between young women with stroke and an age and education matched control group of participants (without stroke) upon completion of SMART.
- To explore the acceptability, appropriateness, and feasibility of SMART in young women with stroke.

## 2 Methods

### 2.1 Design

The study design was a pre-post two group, quasi-experimental pilot research design. The groups consisted of (1) women with stroke and (2) age and education matched controls. All study procedures were approved by the institutional review board and all participants provided written informed consent.

### 2.2 Participants

Participants were recruited by snowball sampling and word of mouth, primarily by outreach to stroke support groups that encompassed younger individuals with stroke. Participants resided in the United States of America in various cities, due to the virtual nature of the study. Participants were recruited during the first four of the 6 months of data collection, which spanned approximately from January to April 2024. See Table 1 for study inclusion and exclusion criteria.

**Table 1:** Participant inclusion and exclusion criteria.

**Participant group**	**Inclusion criteria**	**Exclusion criteria**
Participants with stroke	• Females aged 18–50 years • Sustained a stroke or cerebrovascular accident (CVA) greater than 6 months ago (self-report) • Dwell in the community • Self-reported cognitive challenges	• Severe global aphasia
Control group	• Females aged 18–50 years old	• See below
Both groups	• Willing to participate in all program sessions virtually for duration of program • Consistent Internet access to attend sessions	• Males • Uncorrected vision or hearing loss • Unable to communicate in English • Health condition beyond stroke that impacted cognitive function

### 2.3 Procedures

All study procedures were completed via Zoom. The study included two types of procedures–assessments (pre- and post- SMART) and training.

Assessment: Assessments were independent of SMART and were conducted pre- and post- SMART: All participants were evaluated individually. Pre-SMART assessments took place 1–2 weeks prior to training and post-SMART assessments took place within 1–2 weeks after completion of SMART. Assessment responses were recorded via Zoom^®^ with chat and/or screen share to ensure accuracy. Scoring for the executive function subtests was completed by a trained rater who was independent of the data collection process. Assessments were chosen to encompass multiple aspects of daily life with attention to the feasibility of the program. Assessments included measures of executive function (subtests of the BrainHealth Index), daily living skills (Cognitive Self-Efficacy Questionnaire, Daily Living Questionnaire, and Community Integration Questionnaire), and psychosocial functioning (Depression, Anxiety, and Stress Scale-21), as well as feasibility/goodness of fit of the program 1) Acceptability of Intervention Measure (AIM), Intervention, Appropriateness Measure (IAM), and Feasibility of Intervention Measure (FIM). See Table 2 for assessment details.

**Table 2:** Study assessments.

**Aims**	**Assessments**	**Addresses**
Executive function	Executive Function Subtests^*^ (from the BrainHealth Index) (Center for Brain Health, 2023) **1) Picture themes** **2) Proverb interpretation** **3) Visual selective learning** **4) Test of strategic learning (TOSL)** ^*^2 alternate versions were used for each subtest, randomized between participants. Each participant was presented with a different version of the subtests for their pre- vs. post-assessment.	**1) Picture themes** Addresses abstraction/fluency **2) Proverb interpretation** Addresses abstraction **3) Visual selective learning** Addresses strategic memory: focus on prioritized/important information and inhibition of less important information **4) Test of strategic learning (TOSL)** Addresses executive function. Summary Cohesion–ability to express key information Summary Coherence–ability to express higher level abstract ideas Generation of Highest Lessons–ability to generalize high-level take-home messages Detail Level Recall–ability to recall key information
Daily living skills	**1) Daily living questionnaire (DLQ)** (Toglia, 2004) **2) Cognitive self efficacy questionnaire (CSEQ)** (Toglia and Johnston, n.d.) **3) Community integration questionnaire (CIQ)** (Willer et al., 1993)	**DLQ:** perceived level of cognitive difficulty on various life tasks **CSEQ:** self confidence in complex and everyday cognitive tasks **CIQ:** integration into daily tasks involving others and/or leaving the home
Psychosocial function	**Depression anxiety and stress scale (DASS-21)** (Lovibond and Lovibond, 1995)	Self-report measure examining short term negative emotional states of depression, anxiety, and stress
Feasibility	**1) Acceptability of intervention measure (AIM), intervention Appropriateness measure (IAM), Feasibility of intervention measure (FIM)** (Weiner et al., 2017) **4) Interview**	Goodness of fit for an intervention in a group or population

At the end of SMART and testing, a five question semi-structured interview developed by the principal investigator and validated by experts trained in SMART was administered to each participant with stroke. The goal of this interview was to supplement the summative assessment data, determine positive and negative perceptions of the program, and record individualized perceived impact of the program on the daily lives of the participants. The interview analysis was completed using content analysis to identify themes exploring the feasibility of SMART in this population.

Training: Once the participants were enrolled and assessed, SMART was conducted in real time via Zoom. Participants with stroke were assembled into two groups of four and matched controls completed an identical format of group and training set up. Each group of four (whether stroke or control) remained a distinct group and was not mixed with other groups. Each group completed 10 pre-planned and scheduled 1-h training sessions over 5 weeks, typically meeting with their assigned group and facilitator (the principal investigator) twice a week. PowerPoint^®^ presentations outlining core content with discussion prompts were shared with the group via screen share feature on Zoom. Participants used the chat function and/or verbalized their answers to contribute to the discussions.

Core concepts and principles of the SMART program were introduced in the first 3–4 group sessions. Additionally, during these sessions, participants identified a goal relevant to her daily life, demonstrating practical application of the SMART strategies. In the remaining 5–6 sessions, SMART strategies were integrated and applied in a systematic manner through occupational therapy domains. These included daily living scenarios, integration into daily schedules, role of SMART in health management, importance of SMART in stress management, rest and sleep, and self-care. The final session involved participants sharing the progress toward the goal on which they had been working since the beginning of the study. Groups were designed to be highly interactive, with a strong emphasis on discussion, while the principal investigator served as a facilitator and mentor. All participants with stroke achieved 100 percent attendance for both assessment and group SMART sessions. Among the control group, 7 of 8 control participants also achieved 100 percent attendance with assessment and group SMART sessions, while one participant achieved 90 percent of these sessions.

### 2.4 Analysis

Participant demographics were summarized using measures of central tendency (see Table 3 for details). Mean scores and standard deviations for both the stroke and control groups were reported pre- and post- SMART (see Table 4). Data were analyzed using Microsoft Excel^®^ (Microsoft Office) and IBM^®^ SPSS^®^ Statistics Version 28.0.1.0 (142). Given the small sample size (*n* = 16), non-parametric inferential analyses methods (Wilcoxon signed-rank test for pre and post analysis and Mann Whitney U test for between group analysis) were employed for each of the standardized outcome measures (except for the feasibility metrics). Effect size (*r* statistic, (Rosenthal's *r*), calculated by *Z* score of each test/square root of sample size) was calculated for each statistical test, with effect sizes of 0.1 interpreted as low, 0.3 as medium, and 0.5 or higher as large (Cohen, 1992). Analysis employed a one tailed hypothesis with a *p-*value set at 0.05. Improvements in scores were considered statistically significant if the obtained *p-*value for each outcome measure analysis (when accounting for the unidirectional nature of each hypothesis) was less than 0.05. *Post hoc* corrections and an a priori power analysis were not applied given the pilot nature of the study and its focus on feasibility of the program in a particular population. The AIM, IAM and FIM measures, with data gathered only for the stroke group post SMART, were analyzed descriptively (see Table 4).

**Table 3:** Participant demographics.

**Subject**	**Age (years)**	**Education level**	**Time since stroke (years)**	**Living situation**	**Cognitive challenges**	**Control match**	**Control age**	**Control education level**
S-01	43	Masters	2.75	Alone	Executive function, processing, attention	C-01	42	Masters
S-02	26	Bachelors	2.5	Spouse	Memory, executive function, processing speed	C-06	26	Bachelors
S-05	49	Bachelors	1.25	Alone	Word finding, attention	C-04	43	Bachelors
S-07	39	Masters	2.5	Alone	Attention, processing speed, dual tasking	C-09	39	Masters
S-10	30	Bachelors	5	Parents	Attention, word finding	C-03	26	Bachelors
S-14	32	High school diploma	1.25	Alone	Attention, processing, dual tasking, executive function	C-08	27	High school diploma
S-16	41	Masters	2.75	Alone	Word finding, attention	C-12	37	Clinical doctorate
S-19	50	Masters	3.33	Mother	Word finding, processing, executive function	C-11	46	Masters

**Table 4:** Assessment means and standard deviations.

**Measure**	**Stroke pre SMART *M* (SD)**	**Stroke post SMART *M* (SD)**	**Control pre SMART *M* (SD)**	**Control post SMART *M* (SD)**
**Aim: executive function**				
Picture themes	3 (1.07)	4.38 (1.69)	5.38 (3.07)	6.25 (1.67)
Proverb interpretation	3.38 (2.33)	4.63 (1.06)	4.63 (1.92)	5.13 (0.83)
Visual selective learning	11.13 (4.55)	13.25 (4.62)	13.13 (2.64)	14.88 (3)
**Test of strategic learning:**				
Summary cohesion	4.25 (1.58)	3.88 (1.55)	4.63 (1.19)	4.75 (1.04)
Summary coherence	4 (2.33)	4.25 (3.28)	4.5 (1.41)	4.13 (0.99)
Generation of highest lessons	3.5 (1.85)	5.25 (1.04)	5.5 (0.53)	5.63 (0.52)
Detail level recall	11.88 (5.06)	11.25 (3.69)	14.38 (5.24)	13.50 (4.81)
**Aim: daily living skills**				
**DLQ** ^**^				
Household tasks^*^	1.55 (0.62)	1.46 (0.48)	1.56 (0.49)	1.39 (0.42)
Language and comprehension^*^	1.99 (0.34)	1.94 (0.47)	1.47 (0.49)	1.35 (0.33)
Community participation^*^	1.48 (0.59)	1.44 (0.55)	1.25 (0.38)	1.25 (0.33)
Complex tasks^*^	1.73 (0.39)	1.85 (0.39)	1.6 (0.49)	1.40 (0.35)
Memory^*^	1.69 (0.5)	1.71 (0.74)	1.69 (0.44)	1.53 (0.49)
Executive function monitoring^*^	2.12 (0.92)	1.85 (0.59)	1.4 (0.34)	1.46 (0.47)
Executive function^*^	2.07 (0.55)	1.84 (0.6)	1.56 (0.48)	1.48 (0.51)
**CSEQ**				
Part 1	115.88 (28.28)	129.38 (23.75)	122.88 (15.92)	136.88 (16.75)
Part 2	131 (44.31)	152 (23.92)	149.25 (20.46)	166.38 (20.95)
Part 3	44.38 (8.78)	49.88 (4.42)	48.88 (4.42)	52.38 (5.37)
Part 4	182 (28.79)	192.88 (17.08)	198 (33.87)	220.25 (19.57)
Total	473.25 (103.49)	524.13 (62.94)	519 (66.63)	575.88 (56.56)
**CIQ**				
Home integration	6.84 (2.31)	6.41 (2.54)	6.91 (2.09)	6.94 (2.31)
Social integration	8.5 (2.07)	9.13 (1.73)	9.88 (1.25)	9.88 (1.36)
Productivity	4.13 (1.55)	3.75 (1.83)	6.13 (0.35)	6.13 (0.35)
Total	19.47 (4.14)	19.28 (4.31)	22.91 (2.9)	22.94 (2.83)
**Aim: psychosocial function**				
**DASS-21:**				
Depression^*^	14 (15.46)	8.75 (15.04)	6.5 (11.25)	4.25 (4.46)
Anxiety^*^	4.25 (6.71)	4.25 (4.95)	3.5 (7.62)	1 (2.14)
Stress^*^	14.25 (12.89)	10.25 (9.59)	11.5 (9.61)	9 (6.76)
Total^*^	32.5 (31.02)	23.25 (27.83)	21.5 (27.63)	14.25 (10.71)

^*^Lower score indicates improvement on these measures.

^**^Indicates statistically significant group mean differences at baseline.

The post SMART interviews were analyzed using content analysis (Kleinheksel et al., 2020). The principal investigator conducted the interviews, which were recorded and transcribed. The principal investigator then re-read the transcriptions several times, assigned codes to text, and assigned themes from codes. During this process, three main themes emerged. A second researcher reviewed the data and validated these findings. The key themes that emerged supported the quantitative feasibility findings of the study.

## 3 Results

Sixteen participants completed the study (see Table 3). The participants consisted of two groups of women: (a) eight women with stroke between the ages of 18–50 years (*M* = 38.75, *SD* = ± 8.78), and (b) eight age and education matched control participants (*M* = 35.75, *SD* ± 7.71). Recruitment strategies aimed to match the ages of the control participants to those of the participants with stroke as closely as possible, with an average of 3 years' age difference between each participant with stroke and her age/education matched control. The participants with stroke had a mean time since stroke of 2.67 years (*SD*: ± 1.19).

Group means and standard deviations were calculated on each appropriate outcome measure (see Table 4). While the control group performed slightly better at baseline measurement than the stroke group, only the DLQ scores showed statistically significant differences between the group means at baseline with a large effect size (*Z* = 1.98, *p* = 0.04, *r* = 0.5). Executive function subtests, CSEQ, and community integration/psychosocial measures did not demonstrate statistically significant baseline differences between group means and did not demonstrate large effect sizes (*Z* = −1.79, *p* = 0.07, *r* = 0.45; *Z* = −0.42, *p* = 0.67, *r* = 0.1; *Z* = 0.26, *p* = 0.8, *r* = 0.07, respectively).

### 3.1 Executive function

**Figure 1:**
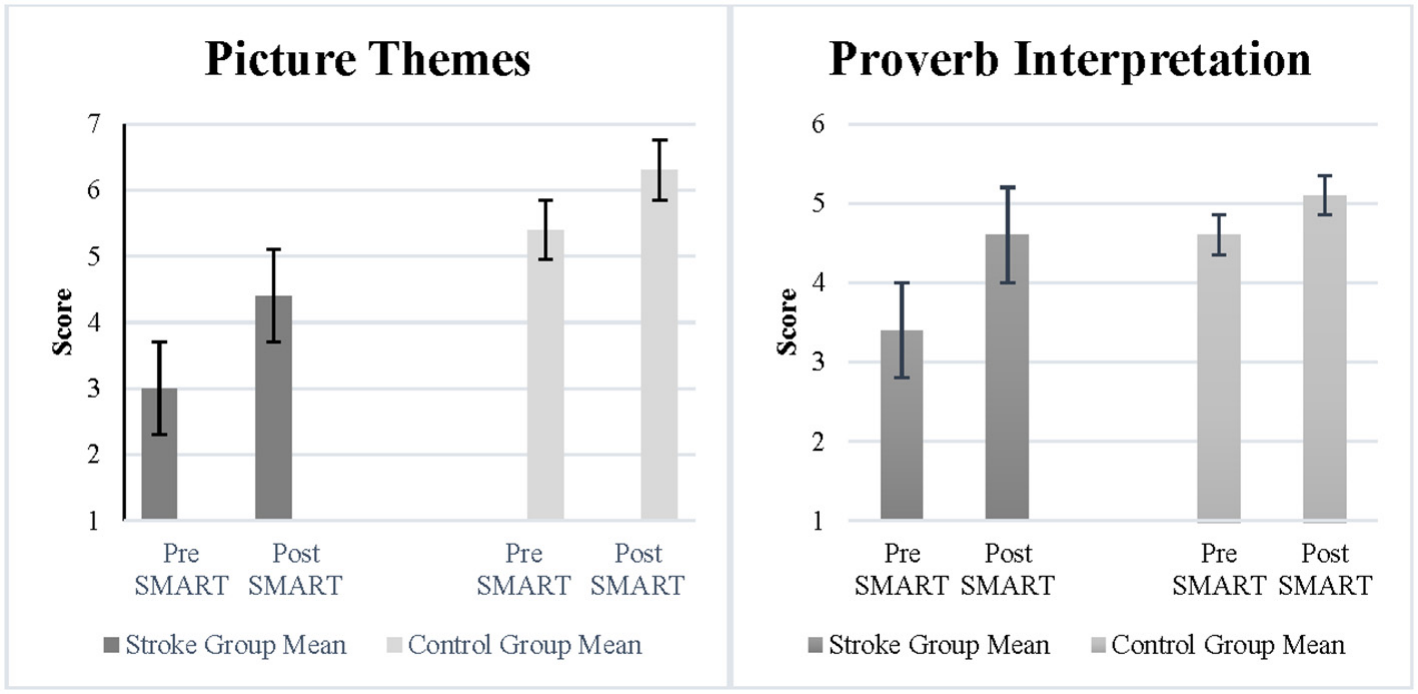
**(A)** Picture themes and **(B)** proverb interpretation scores in stroke and control groups.

The Picture Themes subtest measured abstraction and fluency of ideas. Results demonstrated a statistically significant improvement and a large effect size following SMART as compared to pre SMART for participants with stroke (*Z* = −1.71, *p* = 0.04, *r* = 0.6) (See Figure 1). However, the improvements between the stroke group and the control group were not statistically significant (*Z* = −0.32, *p* = 0.4, *r* = 0.08). The Proverb Interpretation subtest measured verbal abstraction. Results demonstrated a statistically significant improvement and large effect size post SMART as compared to pre SMART for participants with stroke (*Z* = −1.8, *p* = 0.04, *r* = 0.64) (See Figure 1). However, results did not demonstrate a statistically significant improvement or large effect size in change scores between the stroke group and the control group (*Z* = −1.02, *p* = 0.16, *r* = 0.26).

The study used the Visual Selective Learning subtest to measure strategic memory. Results demonstrated a statistically significant improvement and a large effect size in the stroke group (*Z* = −1.78, *p* = 0.04, *r* = 0.63). Additionally, results did not demonstrate statistically significant improvements or large effect sizes in change scores between the stroke group and the control group (*Z* = −0.11, *p* = 0.48, *r* = 0.02).

TOSL measured participants' recall of information and executive function. Results demonstrated a statistically significant improvement with a large effect size for the stroke group following SMART as compared to pre SMART in the Generation of Highest Lessons (*Z* = –1.86, *p* = 0.03, *r* = 0.66). Results did not demonstrate statistically significant improvements or large effect sizes in the stroke group in the remainder of the subtests (Summary Cohesion: *Z* = –0.75, *p* = 0.23, *r* = 0.27; Summary Coherence: *Z* = –0.25, *p* = 0.40, *r* = 0.09; Detail Level Recall: *Z* = 0.0, *p* = 0.5, *r* = 0.0). Additionally, results did not demonstrate a statistically significant improvement in change scores or a large effect size in all measures between the stroke group and the control group (Summary Cohesion: *Z* = –0.87, *p* = 0.22, *r* = 0.02; Summary Coherence: *Z* = –0.27, *p* = 0.4, *r* = 0.06; Generation of Highest Lessons: *Z* = –1.63, *p* = 0.08, *r* = 0.07; Detail Level Recall: *Z* = –0.11, *p* = 0.48, *r* = 0.03).

### 3.2 SMART and daily living skills

**Figure 2:**
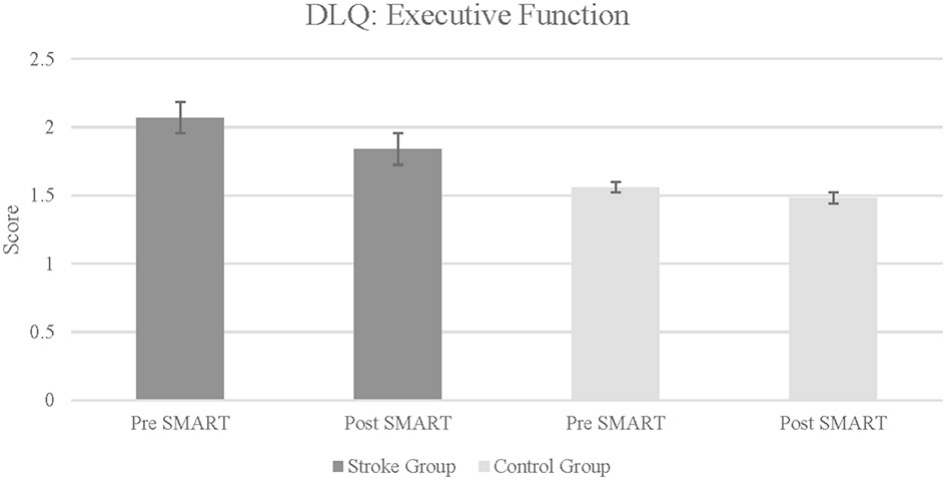
Daily living questionnaire: executive function subtest in stroke and control groups. Lower score indicates improvement in daily living skills and executive function.

DLQ scores in the stroke group revealed a statistically significant improvement in the subtest of Executive Function post SMART as compared to pre SMART, with a large effect size (*Z* = –0.18, *p* = 0.03, *r* = 0.64) (see Figure 2). Results did not demonstrate statistically significant improvements or large effect sizes in subtests of Household Tasks (*Z* = –0.54, *p* = 0.29, *r* = 0.19), Language Comprehension (*Z* = –0.85, *p* = 0.2, *r* = 0.3), Community and Participation (*Z* = –0.67, *p* = 0.25, *r* = 0.24), Complex Tasks: (*Z* = –0.21, *p* = 0.12, *r* = 0.42), or Memory (*Z* = –0.21, *p* = 0.42, *r* = 0.07). Results did indicate a large effect size in the subtest Executive Function Monitoring, although no statistically significant improvement was noted (*Z* = –0.14, *p* = 0.08, *r* = 0.5). Additionally, when change scores between groups were compared, results indicated a statistically significant improvement in the subtest of Complex Tasks between stroke participants and controls (*Z* = –2.06, *p* = 0.02, *r* = 0.51), although the improvement was in the control group.

Part-3 of the DLQ consists of seven broad categorical questions asking about self-perceived ability to think and function, changes in roles and responsibilities, and ability to complete necessary and desired tasks since the illness (stroke). The final question inquires how much the illness (stroke) changed a respondent's life. Due to the nature of these questions, this portion of the questionnaire was only administered to participants with stroke. Each categorical response was ranked and assigned a whole number value (1 through 5 inclusive), with a higher total rank for each question indicating greater level of function. The improvements were found to be statistically significant with a large effect size (*Z* = –2.21, *p* = 0.01, *r* = 0.78).

**Figure 3:**
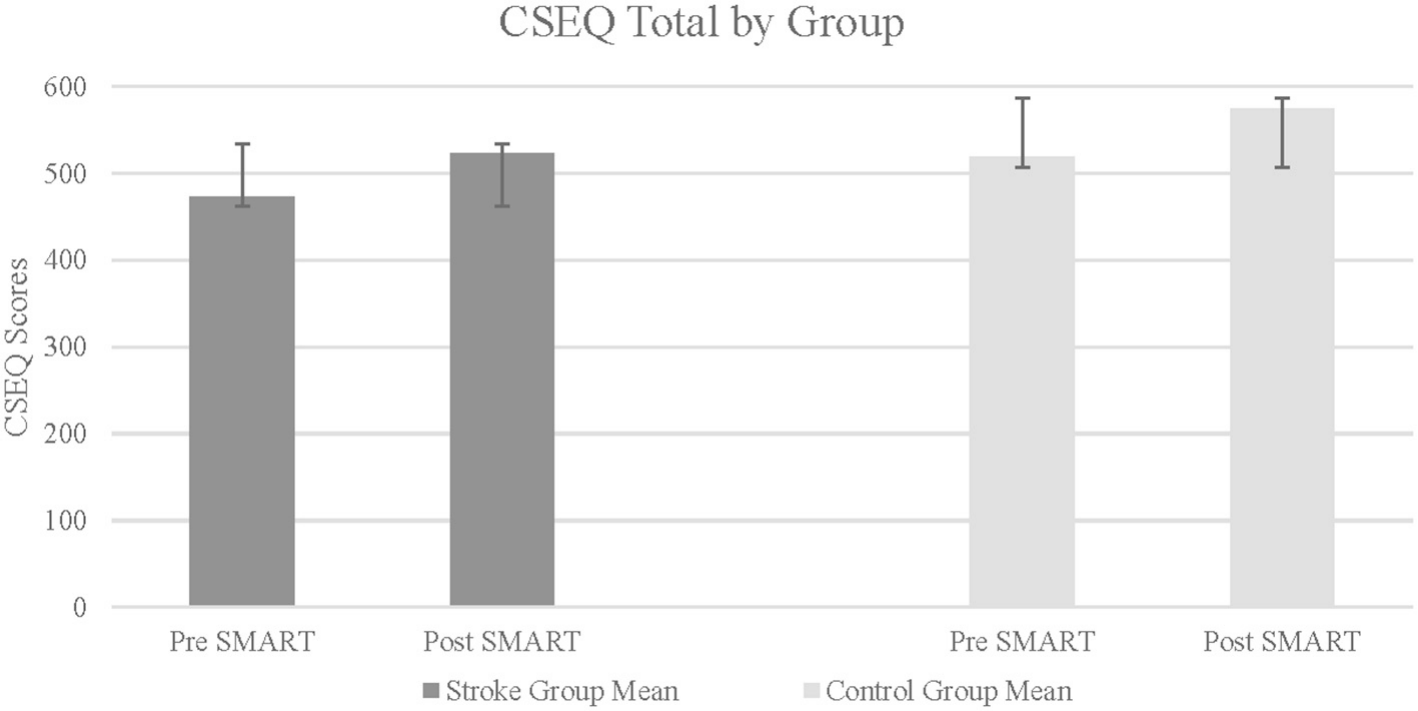
Cognitive self efficacy questionnaire total in stroke and control groups. Higher score indicates greater self efficacy.

CSEQ scores were divided into 4 subtests. For each subtests, as well as for the total CSEQ score, results indicated statistically significant improvements in the stroke group following SMART, as compared to pre SMART, all with large effect sizes (Part 1: *Z* = –2.37, *p* = 0.01, *r* = 0.84; Part 2: *Z* = –1.82, *p* = 0.03, *r* = 0.64; Part 3: *Z* = −2.04, *p* = 0.02, *r* = 0.72; Part 4: *Z* = –1.68, *p* = 0.05, *r* = 0.9; Total: *Z* = –2.24, *p* = 0.01, *r* = 0.79) (see Figure 3). Results did not indicate statistically significant improvements in change scores between groups (Part 1: *Z* = –0.47, *p* = 0.32, *r* = 0.12; Part 2: *Z* = –0.47, *p* = 0.32, *r* = 0.12; Part 3: *Z* = –0.69, *p* = 0.25, *r* = 0.17; Part 4: *Z* = –1.37, *p* = 0.1, *r* = 0.34; Total: *Z* = –0.95, *p* = 0.19, *r* = 0.24).

### 3.3 Community integration and psychosocial function

**Figure 4:**
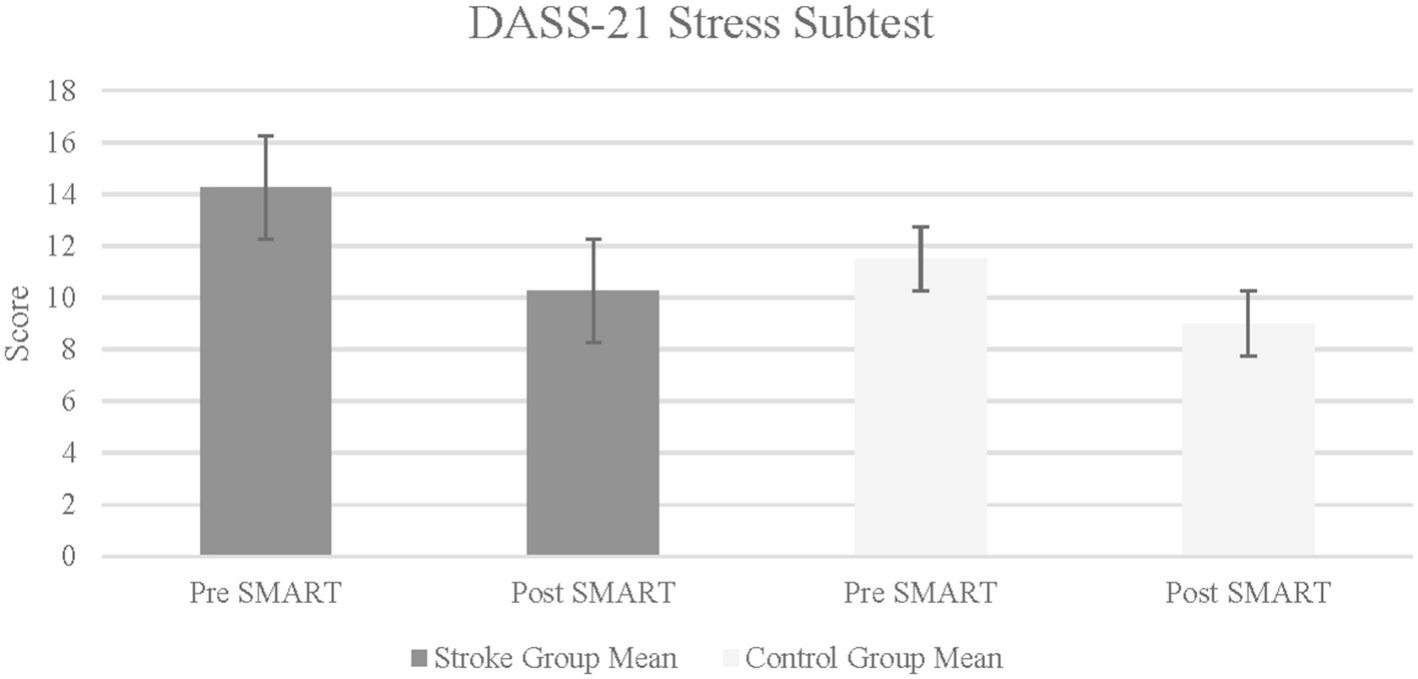
Depression anxiety stress scale-21 in stroke and control groups. Lower score indicates fewer self-reported symptoms of stress.

The results on CIQ and DASS-21 were varied. The stroke group did not indicate statistically significant improvements in any of the subtests of the CIQ (Home Integration: *Z* = –0.82, *p* = 0.21, *r* = −0.29; Social Integration: *Z* = –1.13, *p* = 0.13, *r* = −0.40; Productivity: *Z* = –1.13, *p* = 0.09, *r* = 0.47). Similarly, results did not indicate statistically significant improvements in the change scores of the stroke group as compared to the control group, in any of the CIQ subtests (Home Integration: *Z* = –0.58, *p* = 0.32, *r* = 0.14; Social Integration: *Z* = –1.69, *p* = 0.07, *r* = 0.42; Productivity: *Z* = –1.11, *p* = 0.22, *r* = 0.28). In self-reported symptoms of depression, anxiety and stress (measured using the DASS-21), results indicated statistically significant improvement within the stroke group in Stress subtest from pre- to post- SMART, with a large effect size (*Z* = –1.80, *p* = 0.04, *r* = 0.64; See Figure 4). Results did not indicate statistically significant improvement within the stroke group following SMART as compared to pre SMART on the subtests of Depression (*Z* = –1.29, *p* = 0.99, *r* = 0.46) or Anxiety (*Z* = –0.68, *p* = 0.25, *r* = 0.24). Results did not indicate a statistically significant improvement in change scores between stroke and control groups on any of the subtests (Depression: *Z* = –0.73, *p* = 0.25, *r* = 0.18; Anxiety: *Z* = –1.22, *p* = 0.14, *r* = 0.3; Stress: *Z* = –0.27, *p* = 0.4, *r* = 0.07).

### 3.4 Feasibility

The study employed three feasibility measures (AIM, FIM, IAM) post SMART for participants with stroke. Each four-question measure allows for a range of 4 to 20 points to be achieved, with higher scores reflecting more positive ratings. See Table 5 for details. These generally favorable results were supported by content analysis of the post SMART interviews designed to understand stroke participants' viewpoints on SMART and assist in determining the feasibility of SMART in their lives. Themes that emerged in this content analysis included positive feedback on the use of SMART in daily life, power of group dynamics, and adaptation.

**Table 5:** Acceptability, intervention appropriateness, and feasibility.

**ID**	**Acceptability**	**Appropriateness**	**Feasibility**
S-01	20	19	18
S-05	20	18	20
S-07	16	16	19
S-19	20	20	20
S-02	19	19	17
S-10	12	12	16
S-14	20	20	18
S-16	18	16	17
**Average**	18.13	17.5	18.13

## 4 Discussion

The current study elucidates the potential benefits of the SMART program in mitigating executive function challenges and facilitate participation and engagement in daily life tasks for young women with stroke in chronic stages. While the improvements demonstrated by those in the stroke group were not statistically greater than those demonstrated by the matched control group, the data nonetheless support meaningful clinical benefits, particularly for a potentially under studied population. These findings extend the well-established body of knowledge regarding the efficacy of the SMART program for both neurologically impaired and neurologically intact populations (Vas et al., 2018; Cook et al., 2020; Vas et al., 2020; Chapman et al., 2021; Young et al., 2021).

The generalizable, yet customizable, nature of the SMART program supports meta-cognition, which is a powerful tool enabling constant examination of performance and management of potentially challenging daily scenarios. This self-awareness fosters adaptability and allows the individual using SMART to fine tune these executive functions to meet the dynamic nature of daily experiences. One participant in this study spoke to this concept, stating that awareness and introspection as well as re-evaluation of cognitively taxing aspects of life were key benefits she gained from participation in the study.

Improvements in the DLQ demonstrate the broader impact of SMART in daily life function. Ability to prioritize various tasks while focusing on bigger picture goals contributes to increased mental efficiency and facilitates desired participation in meaningful daily activities requiring these skills. The improvements in overall daily function were further observed in the stroke group, particularly in Part 3 of the DLQ, which examines broader effects of stroke on daily life.

Cognitive self-efficacy, a critical component of meta-cognition, also improved following SMART. Positive effects of improved self-efficacy may include greater adaptation to functional challenges, such as management of unexpected problems, planning, properly managing cognitive fatigue, navigating unfamiliar environments, maintaining compliance with a medication schedule, and completing tasks in a timely manner. Improved self-efficacy can contribute to better self-management and ongoing future improvements in daily life (Nott et al., 2019).

Perception of stress, known to negatively affect executive function (Starcke et al., 2016; Feller et al., 2020), also improved among stroke participants. The strategic attention strategy, a core SMART strategy, can be a powerful tool in minimizing stress. Improvements in stress may also be related to improved self-confidence in navigating multiple daily scenarios, and/or group participation with individuals who share similar life experiences. Regardless, a reduction in stress has broader implications for overall psychosocial wellbeing and is an established benefit of SMART (Young et al., 2021; Laane et al., 2023).

### 4.1 Feasibility considerations

The feasibility of the program is notable in three areas:

1) Clinical and statistical significance: Large effect sizes observed in a small sample size, accompanied by statistical significance, highlight the clinical relevance of the findings. These effect sizes can help to inform future adequately powered trials in the same population.
2) Participant perspective: Overall, participants with stroke provided favorable feedback through interviews regarding the goodness of fit for this intervention. Participants reported consistent use of SMART strategies in the workplace, in the home setting, and in the ability to work toward goals that were challenging since the stroke.
3) Method of delivery: The nature of a pilot study requires that the researchers must balance both scientific rigor and sound methodology while at the same time integrating clinical significance and feasibility into the design.

The SMART program was perceived as valuable in two ways: use of group setting and virtual method of delivery. Participants reported feelings of isolation within the post stroke community, often due to poor alignment of self-identity with others (older adults) with strokes. Participants also reported frustration at frequent societal disbelief regarding the occurrence of their strokes, and the group setting provided validation for their experiences. The power of the group setting, where participants could share common experiences, and provide helpful feedback to one another in a structured yet inviting fashion, seemed to be one of the most salient and highly regarded aspects of the study. It is plausible that the mechanism by which the training was delivered may in fact have influenced its efficacy, given the curated, customized nature of the group approach and the ability of participants to share with each other.

### 4.2 Clinical implications

Virtual program administration promotes desired flexibility within a younger and largely technology-familiar population and increases access points to individuals who may have limited resources for rehabilitation. Additionally, consistent long term follow up as well as recommendations for home programs are feasible for both clinicians and clients in the virtual format.

A second consideration is the importance of screening for executive function deficits in young women with stroke. First, findings from this study demonstrate clinical applicability as they help bring about awareness of potential persistent deficits in young women with stroke. Chronic executive function deficits may be overlooked given the common perception that stroke at a young age is linked to improved outcomes. Routine screenings in this population for executive function deficits as part of regular medical or rehabilitation/therapy visits may help bring to light challenges in these skills that persist in chronic recovery and in turn allow healthcare professionals to make more salient recommendations for treatment and rehabilitation of such deficits.

## 5 Limitations and conclusion

This pilot study was motivated by strong existing evidence of SMART's efficacy in other populations, with the current study representing a necessary first step to explore translation to young women with stroke in chronic stages. Due to the pilot nature of the study, it may be challenging to address other possible factors that could have contributed to gains reported. None of the participants with stroke had children, which is not reflective of the larger population of women in this age group. The outcome measures related to feasibility, daily living and cognitive self-efficacy were self-report measures; future studies should consider performance-based measures. The absence of an interaction analysis (group x time) limits the interpretation of the intervention effect difference between the groups. It is also worth recognizing that use of one-tailed tests without adjustments for multiple comparisons may increase the risk of Type 1 error in the analysis. In this context, findings should be interpreted cautiously and as hypothesis-generating rather than confirmatory. Additionally, the pilot nature of the study and small convenience sample did not feasibly allow for power analysis a priori. Effect size estimates from this study will inform future adequately powered clinical trials. Additionally, all measures were completed via video conferencing online platform, which is a different approach to more established methods of measurement (i.e., in person) and may have influenced outcomes. Finally, investigating a homogeneous group with regards to stroke clinical presentation and impairments could also provide greater insight into the benefits of SMART in this population.

The results of this study contribute to the body of knowledge regarding the feasibility of the telehealth format to administer the SMART program to address executive function in young women with stroke. The study also demonstrates the interconnected nature of functional cognition, with particular attention to the relationship of executive function to daily living skills, self-confidence, social integration, and psychosocial functioning. This study supports the need for examination of current strategies that relate to executive function in young individuals with stroke. In particular, the continued exploration and integration of salient executive function training programs may provide valuable tools for young women with stroke as they navigate an ongoing rehabilitation journey.

## Data Availability

The datasets presented in this article are not readily available because data is available from the corresponding author upon reasonable request. Requests to access the datasets should be directed to mshannahan@twu.edu.
